# Nitrative stress, oxidative stress and plasma endothelin levels after inhalation of particulate matter and ozone

**DOI:** 10.1186/s12989-015-0103-7

**Published:** 2015-09-17

**Authors:** Prem Kumarathasan, Erica Blais, Anushuyadevi Saravanamuthu, Agnieszka Bielecki, Ballari Mukherjee, Stephen Bjarnason, Josée Guénette, Patrick Goegan, Renaud Vincent

**Affiliations:** Analytical Biochemistry and Proteomics Laboratory, Environmental Health Centre, Room 233A, 0803C Tunney’s Pasture, Ottawa, K1A 0 K9 ON Canada; Inhalation Toxicology Laboratory, Environmental Health Science and Research Bureau, Healthy Environments and Consumer Safety Branch, Health Canada, Ottawa, K1A 0 K9 ON Canada

## Abstract

**Background:**

While exposure to ambient air contaminants is clearly associated with adverse health outcomes, disentangling mechanisms of pollutant interactions remains a challenge.

**Objectives:**

We aimed at characterizing free radical pathways and the endothelinergic system in rats after inhalation of urban particulate matter, ozone, and a combination of particles plus ozone to gain insight into pollutant-specific toxicity mechanisms and any effect modification due to air pollutant mixtures.

**Methods:**

Fischer 344 rats were exposed for 4 h to a 3 × 3 concentration matrix of ozone (0, 0.4, 0.8 ppm) and EHC-93 particles (0, 5, 50 mg/m^3^). Bronchoalveolar lavage fluid (BALF), BAL cells, blood and plasma were analysed for biomarkers of effects immediately and 24 h post-exposure.

**Results:**

Inhalation of ozone increased (*p* < 0.05) lipid oxidation products in BAL cells immediately post-exposure, and increased (*p* < 0.05) total protein, neutrophils and mature macrophages in the BALF 24 h post-exposure. Ozone increased (*p* < 0.05) the formation of reactive oxygen species (ROS), assessed by m-, p-, o-tyrosines in BALF (Ozone main effects, *p* < 0.05), while formation of reactive nitrogen species (RNS), indicated by 3-nitrotyrosine, correlated with dose of urban particles (EHC-93 main effects or EHC-93 × Ozone interactions, *p* < 0.05). Carboxyhemoglobin levels in blood exhibited particle exposure-related increase (*p* < 0.05) 24 h post recovery. Plasma 3-nitrotyrosine and o-tyrosine were increased (*p* < 0.05) after inhalation of particles; the effect on 3-nitrotyrosine was abrogated after exposure to ozone plus particles (EHC-93 × Ozone, *p* < 0.05). Big endothelin-1 (BET-1) and ET-1 were increased in plasma after inhalation of particles or ozone alone, but the effects appeared to be attenuated by co-exposure to contaminants (EHC-93 × Ozone, *p* < 0.05). Plasma ET levels were positively correlated (*p* < 0.05) with BALF m- and o-tyrosine levels.

**Conclusions:**

Pollutant-specific changes can be amplified or abrogated following multi-pollutant exposures. Oxidative and nitrative stress in the lung compartment may contribute to secondary extra-pulmonary ROS/RNS formation. Nitrative stress and endothelinergic imbalance emerge as potential key pathways of air pollutant health effects, notably of ambient particulate matter.

**Electronic supplementary material:**

The online version of this article (doi:10.1186/s12989-015-0103-7) contains supplementary material, which is available to authorized users.

## Background

Episodic increases in ambient air contaminant levels have clearly been associated with increased respiratory and cardiovascular morbidity and mortality [1–3]. Traffic-related air pollution affects the autonomic control of the heart and decreases heart rate variability, an indicator of cardiac risk [4]. Air pollution has now been implicated in diverse health impacts such as stroke, Alzheimer’s pathology, mood disorders, gastrointestinal disorders, low infant birth weight and cancer [5–10]. Notwithstanding the strong evidence for adverse health impacts of ambient air pollutants, there remain important knowledge gaps in our understanding of the toxicity mechanisms and the biological plausibility of adverse health outcomes [11]. Notably, direct experimental investigations of contaminant interactions are scarce, and disentanglement of the effects of contaminants inhaled as mixtures in population studies must rely on statistical filters. Understanding of the toxicodynamics of ambient air pollutants and their interactions should allow the development of risk estimate models grounded on direct evidence.

Air pollution is a complex matrix of gases and particulate matter with highly variable physico-chemical characteristics depending on generation mode and sources e.g. point industrial sources, automotive combustion, natural processes such as wild fires and volcanic eruptions, atmospheric conditions. Reactive gases such as ozone, oxides of nitrogen, carbon monoxide, sulphur dioxide and particulate matter of varying size modes (e.g. PM10, PM2.5, ultrafine particles) constitute the air pollutant mixture. Air particles are known sinks for various organics and a number of inorganic chemicals including physiologically active transition metals [12]. Thus investigations of air pollution exposure-induced toxicity mechanisms have become more challenging due to the complexity of air pollutant mixtures [13].

Free radical species are formed in biological systems during cellular respiration at normal physiological levels and they contribute to cellular signal transduction pathways and host defence mechanisms [14]. However, exposure to external stimuli such as air pollutants can result in an excessive generation of free radical intermediates (reactive oxygen species ROS and reactive nitrogen species RNS) and tilt the oxidant/antioxidant balance in the biological system leading to oxidative stress. Reactive gases such as ozone have been reported to function as pulmonary irritants since they can trigger free radical formation that can lead to lung inflammation [15–17]. Squadrito et al., [18] reported that air particles, namely PM 2.5 contained abundant persistent semiquinone radicals. These radicals can contribute to redox cycling reactions *in vivo*. It is therefore feasible that exposure to air pollutants can lead to the formation of reactive free radicals which can trigger or modulate onset of existing pathological conditions.

We have previously shown that exposure of rats to ozone by inhalation can cause the generation of hydroxyl radicals and reactive oxygen species [16, 19, 20]. We have also observed the formation of reactive intermediate-derived metabolites in TNFα-positive transgenic mice exposed by inhalation to a mixture of ozone and air particles for 90 days [21]. Ambient particulate matter and combustion-generated particles including diesel exhaust particles have been shown to contribute to the formation of reactive oxygen species [22, 23]. We have also shown previously that air particle exposures led to increased blood pressure in animals along with increased levels of circulating endothelin ET-1, a potent vasoconstrictor peptide [24]. Endothelin-1 is a known prognostic factor for cardiovascular diseases.

The objectives of this study were to first, identify systematically if there were differences in *in vivo* oxidative stress mechanisms due to the type of air pollutant exposure such as ozone, particulate matter or to mixtures. Secondly, we intended to assess the effect of these air pollutant exposures on the integrity of the vascular endothelium. Thirdly, our goal was to investigate any association between air pollutant-induced oxidative stress and vasoregulatory mechanisms. Finally, our aim was to analyse for inter-pollutant interactions and recovery time-related effects. The main goal was to investigate if oxidative stress and vascular dysfunction-related pathways can permit disentanglement of effects caused by individual pollutants within the air pollutant mixtures. In order to achieve these objectives, we exposed male Fisher 344 rats either to ozone, ambient air particles or various mixtures of these pollutants and measured various relevant biological endpoints at immediately and 24 h post-exposure.

## Results

### Lung injury markers

**Fig. 1:**
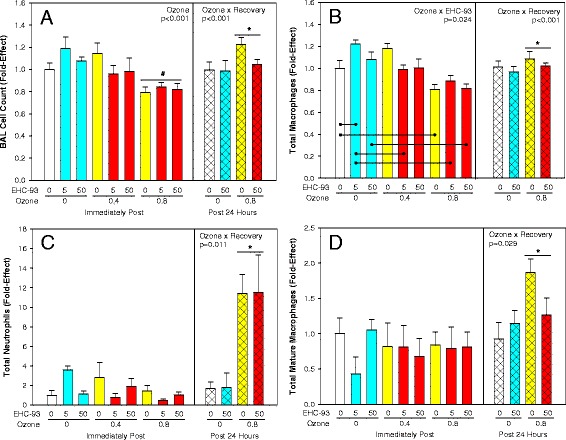
Bronchoalveolar lavage cytology. Results expressed as mean fold-effect ± SEM. **a**. Total cell count. 3-way ANOVA, Ozone x Recovery, *p* < 0.001. Tukey, *0 vs 0.8 ppm O_3_ within 24 h recovery, and 0 h vs 24 h recovery within 0 and 0.8 ppm O_3_, *p* < 0.05. 2-way ANOVA, Ozone, *p* < 0.001. Tukey, ^#^0 and 0.4 vs 0.8 ppm O_3_, *p* < 0.05. **b**. Total macrophages count. 3-way ANOVA, Ozone × Recovery, *p* < 0.001. *0 vs 0.8 ppm O_3_ within 24 h. 2-way ANOVA, Ozone × EHC-93, *p* = 0.024. Tukey summarized by lines (e.g. 0 vs 5 mg/m^3^ EHC-93 within 0 ppm O_3_), *p* < 0.05. **c**. Total neutrophils count. 3-way ANOVA, Ozone × Recovery, *p* = 0.011 and Ozone × EHC-93, *p* = 0.030. Tukey, *0 vs 0.8 ppm O_3_ within 24 and 0 h vs 24 h within 0.8 ppm O_3_. **d**. Total mature macrophages. 3-way ANOVA, Ozone × Recovery, *p* = 0.029. Tukey, 0 vs 0.8 ppm O_3_ within 24 h. For detailed statistics, refer to Additional files 4 & 6. (Sample size, *n* = 8/air pollutant exposure group; *n* = 17/air control group)

**Fig. 2:**
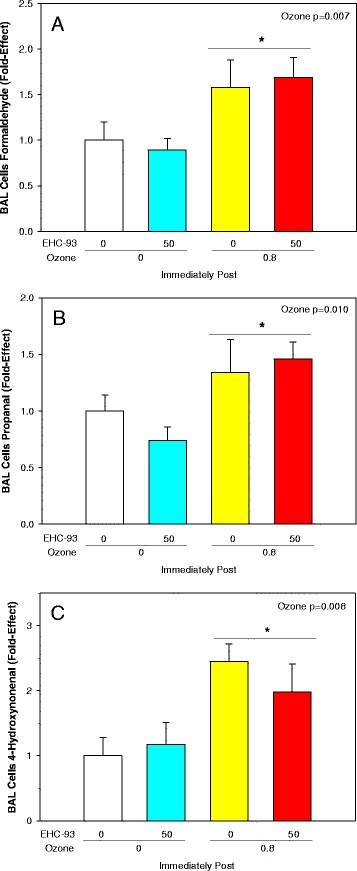
Lipid oxidation products in the BAL cells immediately post-exposure to air pollutants. Mean fold-effect ± SEM. **a**. Formaldehyde. 2-way ANOVA, Ozone, *p* = 0.007. Tukey, *0 vs 0.8 ppm O_3_, *p* < 0.05. **b**. Propanal. 2-way ANOVA, Ozone, *p* = 0.010. Tukey, *0 vs 0.8 ppm O_3_, *p* < 0.05. **c**. 4-Hydroxynonenal. 2-way ANOVA, Ozone, *p* = 0.008. Tukey, *0 vs 0.8 ppm O_3_, *p* < 0.05. For detailed statistics, refer to Additional file 6. (Sample size, *n* = 4/air pollutant exposure group; *n* = 4/air control group)

**Fig. 3:**
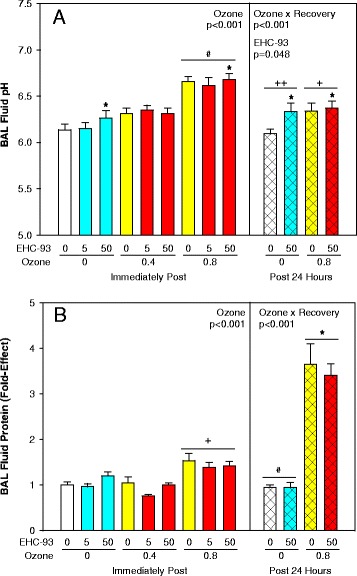
BALF injury markers. **a**. pH of BALF. Mean ± SEM. 3-way ANOVA, EHC-93, *p* = 0.048 and Ozone × Recovery, *p* < 0.001. Tukey, *0 vs 50 mg/m^3^ EHC-93, ^†^0 h vs 24 h within 0.8 ppm O_3_, ^‡^0 h vs 24 h within 0 ppm O_3_, *p* < 0.05. 2-way ANOVA, Ozone, *p* < 0.001. Tukey, ^#^0 and 0.4 vs 0.8 ppm O_3_, *p* < 0.05. **b**. BALF protein. Mean fold-effect ± SEM. 3-way ANOVA, Ozone x Recovery, *p* < 0.001. Tukey, *0 h vs 24 h within 0.8 ppm O_3_, ^#^0 h vs 24 h within 0 ppm O_3_, *p* < 0.05. 2-way ANOVA, Ozone, *p* < 0.001. Tukey, ^†^0 and 0.4 vs 0.8 ppm O_3_, *p* < 0.05. For detailed statistics, refer to Additional files 4 & 6. (Sample size, *n* = 8/air pollutant exposure group; *n* = 17/air control group)

Total BAL cells were decreased by 20 % immediately post-exposure to 0.8 ppm O_3_ but increased slightly 24 h post-exposure (Fig. 1a; 3-way ANOVA, Ozone x Recovery, *p* < 0.001), a pattern of change attributed to the total macrophage population (Fig. 1b; 3-way ANOVA, Ozone × Recovery, *p* = 0.001; 2-way ANOVA, Ozone × EHC-93, *p* = 0.024). Note: Statistical analysis was conducted on rank transformed data for macrophages, neutrophils and mature macrophages to meet the assumptions for normality and equal variance. Neutrophils were increased 12-fold (Fig. 1c; 3-way ANOVA, Ozone × Recovery, *p* = 0.011), while mature macrophages were increased by about 2-fold 24 h post-exposure to 0.8 ppm O_3_, without any effects of particle deposition (Fig. 1d; 3-way ANOVA, Ozone × Recovery, *p* = 0.029). Changes of lipid oxidation products formaldehyde (Fig. 2a; 2-way ANOVA, Ozone, *p* = 0.007), propanal (Fig. 2b; Ozone, *p* = 0.010) and 4-hydroxynonenal (Fig. 2c; Ozone, *p* = 0.008) in total BAL cells were related to inhalation of O_3_ and not particles. Both O_3_ (3-way ANOVA, Ozone x Recovery, *p* < 0.001; 2-way ANOVA, Ozone main effect, *p* < 0.001) and particles (three-way ANOVA, EHC-93 main effect, *p* = 0.048) increased the pH value of the BALF supernatants, immediately after and 24 h after exposure, with apparent simple additive effects of the pollutants (Fig. 3a). Exposure to O_3_ elevated total BALF protein 1.5-fold immediately, and 3.5-fold 24 h after exposure, without any main effects or interaction of particles (Fig. 3b; 3-way ANOVA, Ozone x Recovery, *p* < 0.001; 2-way ANOVA, Ozone, *p* < 0.001).

### BALF oxidative stress markers

**Fig. 4:**
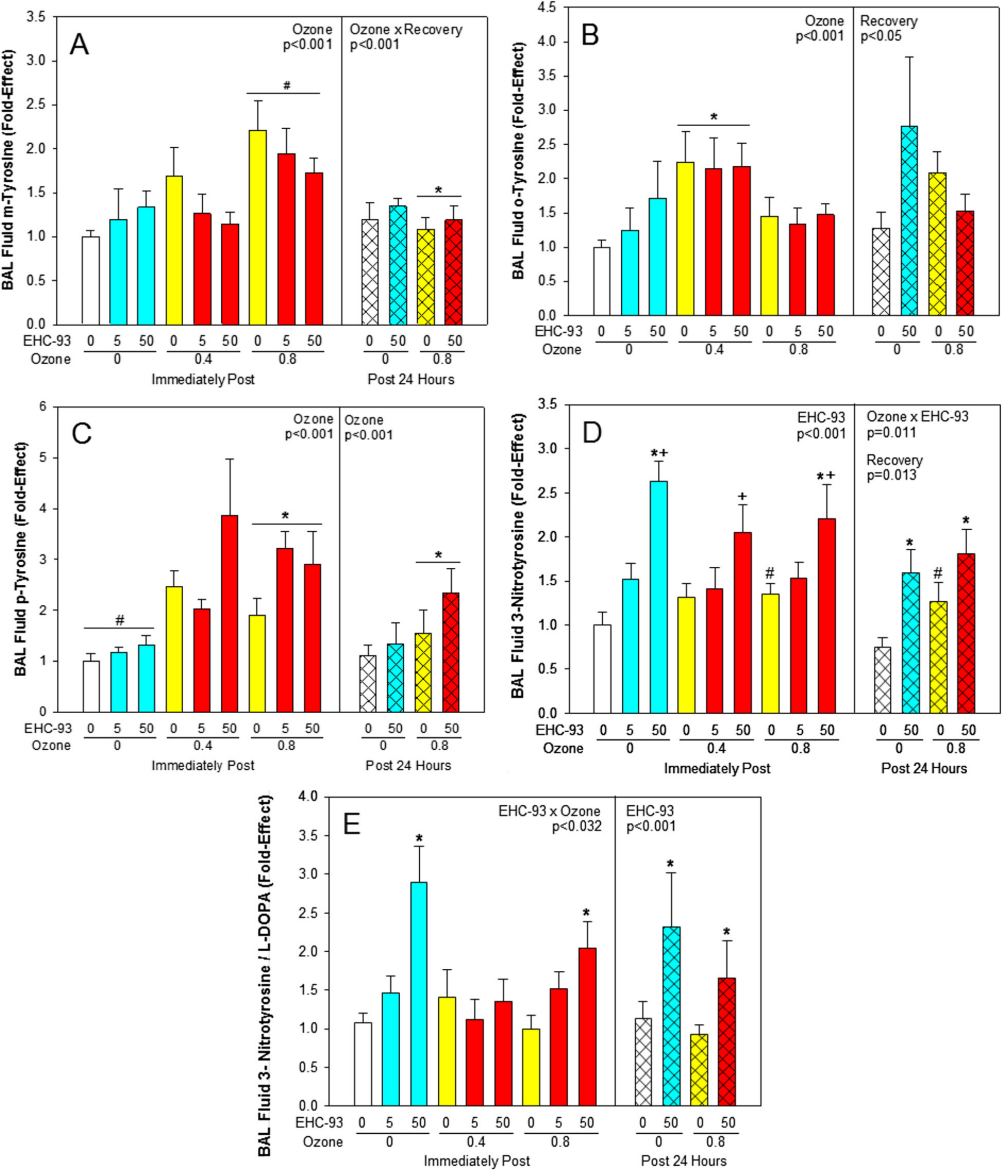
BALF markers of ROS and RNS formation. Mean fold-effect ± SEM. **a**. m-Tyrosine. 3-way ANOVA, Ozone × Recovery, *p* < 0.001. Tukey, *0 vs 0.8 ppm O_3_ within 24 h, *p* < 0.05. 2-way ANOVA, Ozone, *p* < 0.001. Tukey, ^#^0 and 0.4 vs 0.8 ppm O_3_, *p* < 0.05. **b**. o-Tyrosine. 3-way ANOVA, Recovery, *p* = 0.047. Tukey, 0 h vs 24 h, *p* < 0.05. 2-way ANOVA, Ozone, *p* < 0.001. Tukey, *0 and 0.8 vs 0.4 ppm O_3_, *p* < 0.05. **c**. p-Tyrosine. 3-way ANOVA, Ozone, *p* < 0.001. Tukey, *0 vs 0.8 ppm O_3_, *p* < 0.05. 2-way ANOVA, Ozone, *p* < 0.001. Tukey, 0 vs 0.4 and 0.8 ppm O_3_, *p* < 0.05. **d**. 3-Nitrotyrosine. 3-way ANOVA, Recovery, *p* = 0.013 and Ozone × EHC-93, *p* = 0.011. Tukey, 0 h vs 24 h, *0 vs 50 mg/m^3^ within 0 and 0.8 ppm O_3_, ^#^0 vs 0.8 ppm O_3_ within 0 and 50 mg/m^3^, *p* < 0.05. 2-way ANOVA, EHC-93, *p* < 0.001. Tukey, ^†^0 and 5 vs 50 mg/m^3^, *p* < 0.05. **e**. 3-Nitrotyrosine/L-Dopa. 3-way ANOVA, EHC-93, *p* < 0.001. Tukey, *0 vs 50 mg/m^3^ EHC-93, *p* < 0.05. 2-way ANOVA, EHC-93 × Ozone, *p* = 0.032. Tukey, *0 and 5 vs 50 mg/m^3^ EHC-93 within 0 ppm O_3_ and 0 vs 50 mg/m^3^ EHC-93 within 0.8 ppm O_3_, *p* < 0.05. For detailed statistics, refer to Additional files 4 & 6. (Sample size, *n* = 8/air pollutant exposure group; *n* = 15/air control group)

Exposure to O_3_ enhanced (*p* < 0.05) m- o- and p-tyrosine levels in BALF (Fig. 4a–c; 2-way ANOVA, Ozone main effect, *p* < 0.001). Ozone and particles could independently increase 3-nitrotyrosine in BALF, but also with interaction (Fig. 4d; 3-way ANOVA, Ozone x EHC-93, *p* = 0.011, Recovery main effect, *p* = 0.013; 2-way ANOVA, EHC-93 main effect, *p* < 0.001). The ratio of 3-nitrotyrosine to L-DOPA, which provides a ratio of the relative activity of nitration pathways over that of hydroxylation pathways, was increased with exposure to EHC-93 only, both immediately and 24 h post recovery period, with apparent competition of hydroxylation over nitration at 0.4 ppm O_3_ (Fig. 4e; 3-way ANOVA, EHC-93 main effect, *p* < 0.001; 2-way ANOVA, EHC-93 × Ozone, *p* = 0.032). Protein carbonyl levels in BALF were not significantly altered by the pollutants (Additional file 1).

### Hemoglobin

**Fig. 5:**
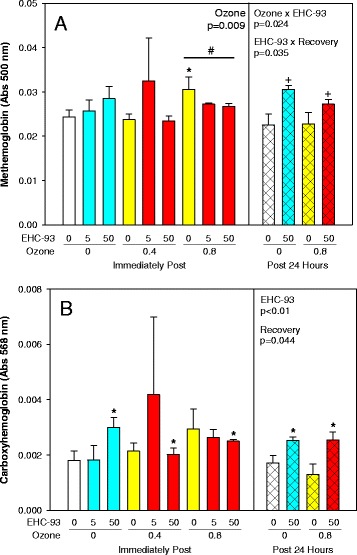
Hemoglobin variants in whole blood. Mean absorbance ± SEM. **a**. Methemoglobin. 3-way ANOVA, Ozone × EHC-93, *p* = 0.024 and EHC-93 × Recovery, *p* = 0.035. Tukey, *0 vs 0.8 ppm O_3_ within 0 mg/m^3^ EHC-93, ^†^0 h vs 24 h Recovery within 50 mg/m^3^ EHC-93, *p* < 0.05. 2-way ANOVA, Ozone, *p* = 0.009. Tukey, ^#^0.4 vs 0.8 ppm O_3_, *p* < 0.05. **b**. Carboxyhemoglobin. 3-way ANOVA, EHC-93, *p* < 0.001 and Recovery, *p* = 0.044. Tukey, *0 vs 50 mg/m^3^ EHC-93, ^†^0 h vs 24 h Recovery, *p* < 0.05. For detailed statistics, refer to Additional files 5 & 7. (Sample size, *n* = 4/air pollutant exposure group; *n* = 6/air control group)

Interestingly, inhalation of both pollutants altered the levels of methemoglobin in a recovery-dependent manner after exposure (Fig. 5a; 3-way ANOVA, Ozone × EHC-93, *p* = 0.024, EHC-93 × Recovery, *p* = 0.035; 2-way ANOVA, Ozone, *p* = 0.009). However, inhalation of particles elevated carboxyhemoglobin even after 24 h post recovery (Fig. 5b; 3-way ANOVA, EHC-93 main effect, *p* < 0.001, Recovery main effect, *p* = 0.044). Furthermore, the levels of oxyhemoglobin and sulfhemoglobin were not affected significantly by these pollutant exposures.

### Plasma oxidative stress markers

**Fig. 6:**
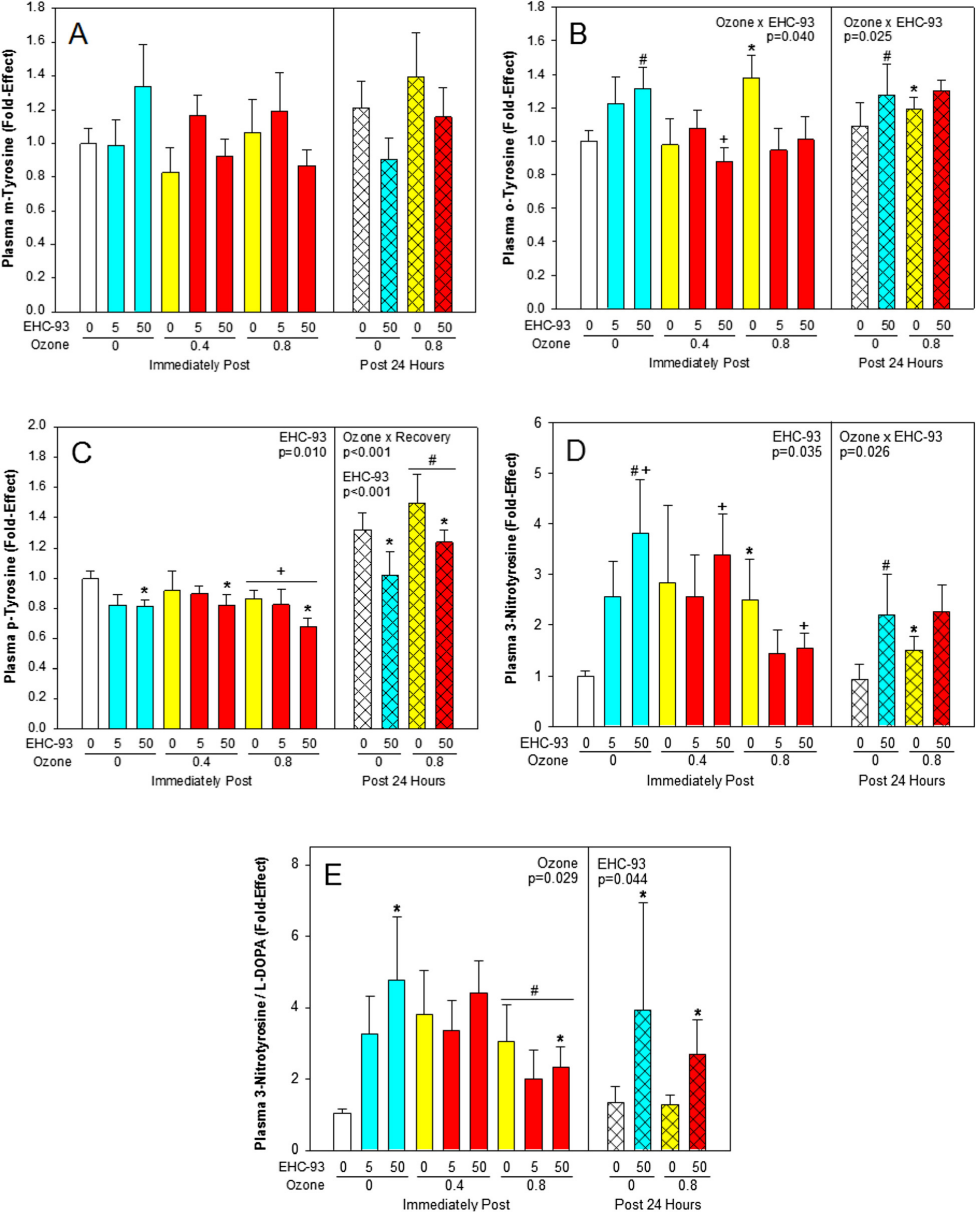
Plasma markers of ROS and RNS formation. Mean fold-effect ± SEM. **a**. m-Tyrosine, not significant. **b**. o-Tyrosine. 3-way ANOVA, Ozone × EHC-93, *p* = 0.025. Tukey, *0 vs 0.8 ppm O_3_ within 0 mg/m^3^ EHC-93, ^#^0 vs 50 mg/m^3^ EHC-93 within 0 ppm O_3_, *p* < 0.05. 2-way ANOVA, Ozone × EHC-93, *p* = 040. Tukey, ^†^0 vs 0.4 ppm O_3_ within 50 mg/m^3^ EHC-93, *p* < 0.05. **c**. p-Tyrosine. 3-way ANOVA, EHC-93, *p* < 0.001 and Ozone × Recovery, *p* < 0.001. Tukey, *0 vs 50 mg/m^3^ EHC-93, ^#^0 vs 0.8 ppm O_3_ within 24 h, ^†^0 vs 0.8 ppm O_3_ within 0 h recovery, *p* < 0.05. 2-way ANOVA, EHC-93, *p* = 0.010. Tukey, *0 vs 50 mg/m^3^ EHC-93, *p* < 0.05. **d**. 3-Nitrotyrosine. 3-way ANOVA, Ozone × EHC-93, *p* = 0.026. Tukey, *0 vs 0.8 ppm O_3_ within 0 mg/m^3^ EHC-93, ^#^0 vs 50 mg/m^3^ EHC-93 with 0 ppm O_3_, *p* < 0.05. 2-way ANOVA, EHC-93, *p* = 0.035. Tukey, ^†^0 vs 50 mg/m^3^ EHC-93, *p* < 0.05. **e**. 3-Nitrotyrosine/L-Dopa. 3-way ANOVA, EHC-93, *p* = 0.044. Tukey, *0 vs 50 mg/m^3^ EHC-93, *p* < 0.05. 2-way ANOVA, Ozone, *p* = 0.029. Tukey, ^#^0.4 vs 0.8 ppm O_3_, *p* < 0.05. For detailed statistics, refer to Additional files 5 & 7. (Sample size, *n* = 7–8/air pollutant exposure group; *n* = 14/air control group)

Changes of plasma m-tyrosine levels after exposure to air contaminants did not reach statistical significance (Fig. 6a), while o-tyrosine (Fig. 6b; 3-way ANOVA, Ozone × EHC-93, *p* = 0.025; 2-way ANOVA, Ozone × EHC-93, *p* = 0.040) and p-tyrosine (Fig. 6c; 3-way ANOVA, Ozone × Recovery, *p* < 0.001, EHC-93 main effect, *p* < 0.001; 2-way ANOVA, EHC-93 main effect, *p* = 0.010) were affected by both pollutants. Inhalation of the particles resulted in a nitrative stress with elevation of 3-nitrotyrosine, a response that was partly abrogated by high concentration of O_3_ (Fig. 6d; 3-way ANOVA, Ozone × EHC-93, *p* = 0.026; 2-way ANOVA, EHC-93 main effect, *p* = 0.035). Ratio of nitration to hydroxylation, indicated by 3-nitrotyrosine over L-DOPA presented a similar pattern, with elevation by EHC-93 and abrogation by O_3_ (Fig. 6e; 3-way ANOVA, *p* = 0.044; 2-way ANOVA, Ozone main effect, *p* = 0.029. The pattern of the lipid oxidation marker 8-isoPGF2α in plasma was similar to that of 3-nitrotyrosine, but changes did not reach significance (Additional file 2).

### Endothelial dysfunction markers

**Fig. 7:**
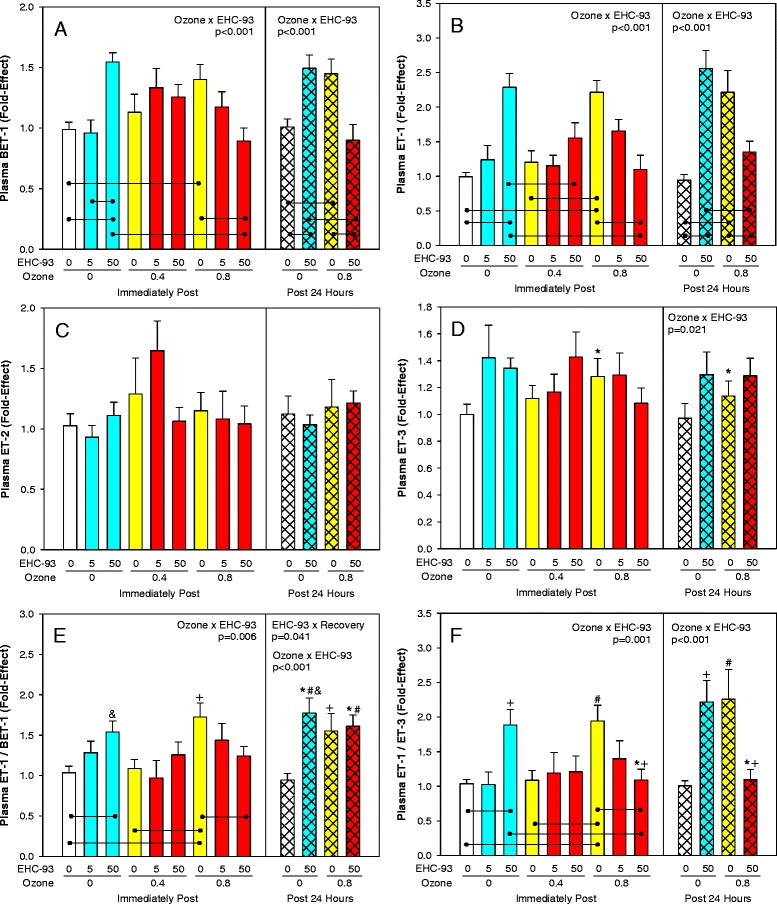
Plasma endothelin isoforms. Mean fold-effect ± SEM. **a**. BET-1. 3-way ANOVA and 2-way ANOVA, Ozone × EHC-93, *p* < 0.001. Tukey multiple comparisons, lines, *p* < 0.05. **b**. ET-1. 3-way ANOVA and 2-way ANOVA, Ozone × EHC-93, *p* < 0.001. Tukey multiple comparisons, lines, *p* < 0.05. **c**. ET-2, not significant. **d**. ET-3. 3-way ANOVA, Ozone × EHC-93, *p* = 0.021. Tukey, *0 vs 0.8 ppm O_3_ within 0 mg/m^3^ EHC-93. E. Ratio ET-1/BET-1. 3-way ANOVA, Ozone × EHC-93, *p* < 0.001 and EHC-93 × Recovery, *p* = 0.041. Tukey, *0 vs 50 mg/m^3^ EHC-93 within 24 h recovery, ^#^0 h vs 24 h within 50 mg/m^3^ EHC-93, ^†^0 vs 0.8 ppm O^3^ within 0 mg/m^3^ EHC-93, &0 vs 50 mg/m^3^ EHC-93 within 0 ppm O_3_, *p* < 0.05. 2-way ANOVA, Ozone × EHC-93, *p* = 0.006. Tukey comparisons, lines, *p* < 0.05. **f**. Ratio ET-1/ET-3. 3-way ANOVA, Ozone × EHC-93, *p* < 0.001. Tukey, *0 vs 0.8 ppm O_3_ within 50 mg/m^3^ EHC-93, ^#^0 vs 0.8 ppm O_3_ within 0 mg/m^3^ EHC-93, ^†^0 vs 50 mg/m^3^ within 0 and 0.8 ppm O_3_, *p* < 0.05. 2-way ANOVA, Ozone × EHC-93, *p* = 0.001. Tukey, lines, *p* < 0.05. For detailed statistics, refer to Additional files 5 & 7. (Sample size, *n* = 7–8/air pollutant exposure group; *n* = 14/air control group)

Analysis of circulating endothelin isoform levels revealed an increase of BET-1 (Fig. 7a) and ET-1 (Fig. 7b) after exposures to either EHC-93 or O_3_, with a negative interaction when inhaled together (3-way and 2-way ANOVA, Ozone × EHC-93, *p* < 0.001). There were no significant changes of the ET-2 isoform in plasma (Fig. 7c), while ET-3 was affected by O_3_ (Fig. 7d; 3-way ANOVA, Ozone × EHC-93, *p* = 0.021). While elevation of BET-1 reflects mainly increased de novo synthesis, the ratio ET-1/BET-1 as an index of the rate of ECE-1 conversion of BET-1 to ET-1 was elevated after exposure to the pollutants (Fig. 7e; 3-way ANOVA, EHC-93 × Recovery, *p* = 0.041, Ozone × EHC-93, *p* < 0.001; 2-way ANOVA, Ozone × EHC-93, *p* = 0.006). The ratio ET-1/ET-3 as an index of ET[A] receptor mediated vasoconstriction and ET[B] receptor mediated vasodilation followed a pattern very similar to that of ET-1 (Fig. 7f; 3-way and 2-way ANOVA, *p* ≤ 0.001). Changes of plasma nitrite levels were not statistically significant (Additional file 3). The levels of m-tyrosine in BALF were positively correlated with circulating BET-1 (Spearman rank order, *r* = 0.330, *p* = 0.033) and ET-1 (Spearman, *r* = 0.309, *p* = 0.047) levels. Plasma o-tyrosine levels were positively correlated with circulating ET-1 (Spearman, *r* = 0.211, *p* = 0.015). Backward stepwise regression analyses indicated that BALF m-tyrosine was a predictor of plasma BET-1 (*p* = 0.028), while a linear combination of BALF m-tyrosine (*p* = 0.015) and plasma BET-1 (*p* < 0.001) were predictors of circulating levels of ET-1.

## Discussion

In this study, we investigated the impact of inhaled ozone, airborne particles and the mixtures on nitrative stress, oxidative stress and circulating endothelin levels in experimental animals. The urban air particle EHC-93 has been used widely as a model particle in air pollutant toxicity investigations [8, 21, 24–26]. As discussed before, while the absolute concentration of the total suspended particulate matter is high relative to PM_2.5_ levels in North America, the alveolar deposited dose of fine particulate mass is within an order of magnitude of realistic human exposure scenario.[24] The elevation of the total number of neutrophils (Fig. 1c) and protein (Fig. 3b) levels in BAL confirmed lung injury attributable mainly to O_3_ [27, 28].

Analysis of BAL cells for oxidized lipids such as formaldehyde, propanal and 4-hydroxynonenal revealed increased generation of lipid oxidation products after exposure to ozone (Fig. 2). This observation can be attributed to reaction of ozone with unsaturated lipids in these cells, potentially via an “ozonide” intermediate that cleaves to form aldehydes and hydrogen peroxide (H_2_O_2_) [16, 29]. These changes were not noticed with animals exposed to EHC-93 alone, even though air particles can contribute to redox cycling-related lipid oxidation. This observation illustrates an ozone exposure-specific biological change at the target site (Fig. 2). The fact that ozone exposure led to lipid oxidation product formation in BAL cells immediately after exposure which was followed by increased BAL neutrophils after 24 h post-exposure suggest that these lipid oxidation products perhaps contributed to defence cell recruitment mechanisms. This is in line with a previous report where intervention with quercertin reduced lipid oxidation product formation in alveolar macrophages and a concurrent reduction in neutrophil infiltration [30]. Similarly, the observation of increased mature macrophages at 24 h post recovery after ozone exposure could be linked to increased lipid oxidation in BAL cells as well. This is supported by previous reports on increased lipid oxidation, foam cell formation and presence of mature macrophages in lungs especially after a lung infection or oxidant insult that is typically associated with inflammatory diseases of the lung [31, 32].

The observation of increased BAL Fluid (BALF) protein levels in animals exposed to ozone as a constituent of the pollutant matrix, after 24 h post recovery suggested compromised cellular membrane integrity (Fig. 3b). Increased BAL neutrophils and BALF protein in the lung suggest inflammation or irritation of lungs when exposed to ozone containing atmospheres as opposed to air particles, when such frank effects are not observed.

We also noticed differences in the kinetics of formation of m-, o- and p-tyrosine isomers immediately and 24 h post-exposure to pollutants, in this study. *In vivo* formation of reactive oxygen species (ROS) notably OH•, has been shown to react with phenylalanine to form these m-, o- and p- tyrosines [33]. p-Tyrosine can also be formed enzymatically. Levels of m-tyrosine in BALF exhibited a clear dose-related increase in ozone exposed animals, immediately after exposure (Fig. 4), whereas, p- and o-tyrosine isomers exhibited non-monotonic changes with increasing ozone dose. Particle exposures appeared to impact on BALF o-tyrosine levels. Ambient air particles host a variety of metal species including transition metals which can favour o-tyrosine formation compared to the other tyrosine isomers by the formation of a relatively stable metal complex intermediate involving the “R (−CH_2_CHNH_2_COOH)” moiety in phenylalanine and the “ortho” position adjacent to it. The corresponding intermediate metal complexes associated with the formation of m- or p- isomers may not be energetically feasible. This notion is consistent with previous reports on formation of o-tyrosine and dityrosine as a result of metal catalyzed oxidation [34]. When exposed to air pollutant mixtures, m-, o- and p-tyrosine levels were modified by the constituents (Fig. 4). Ozone and EHC-93 particles apparently modulated the effects caused by the other. This is revealed by BALF p-tyrosine levels that exhibited additive effect, while o-tyrosine levels showed a suppressive effect, especially at 24 h post-exposure to pollutant mixtures.

3-Nitrotyrosine, a product of tyrosine nitration exhibited a dose-related increase in BALF of EHC-93 exposed animals, which was modulated by the level of ozone in the mixtures (Fig. 4). Reaction of tyrosine with peroxynitrite, a reactive nitrogen intermediate can lead to the formation of 3-nitrotyrosine. Peroxynitrite can be formed as a result of the reaction of superoxide (O_2_^−^•) with NO• or due to a reaction of OH• with NO_2_ or H_2_O_2_ with NO•, at diffusion limited rates [35]. Reactive oxygen species can be generated *in vivo* as a result of ozone or EHC-93 exposures [24, 36]. Meanwhile, EHC-93 particles perhaps can trigger host-defense mechanisms up-regulating inducible nitric oxide synthase iNOS and thus NO• production. Although these particles do not cause pulmonary inflammation, interestingly there was an increasing trend in total BAL cells immediately post exposure when the animals were exposed to particles alone which may have led to increase in iNOS [37] and thus NO• production in lung. Squadrito et al. [18] have shown that when engulfed by BAL cells, particles enhanced NO• production that results in peroxynitrite formation. Furthermore, at physiological conditions, pH has been shown to influence tyrosine nitration [38]. Interestingly, pH values of BALF (Fig. 3) from animals exposed to EHC-93 were relatively low compared to those of ozone exposed animals. Under such conditions, particle exposures presumably lead to the formation of “peroxynitrous acid” which can subsequently undergo heterolytic cleavage to yield “nitronium” ion that nitrates p-tyrosine residues in proteins. It is known that formation of “nitronium” ion from peroxynitrite is favoured in the presence of protein bound or free metal ions [39]. The profiles of 3-nitrotyrosine to L-dopa ratio (Fig. 4) can serve as a measurement of the competition between RNS and ROS-mediated reactions. L-dopa is a hydroxylation product of p-tyrosine and can be formed by reaction with OH•. Our data on 3-Nitrotyrosine to L-dopa ratio values revealed that with EHC-93 exposures, RNS-mediated reaction was favoured compared to ROS-dependent reaction as opposed to exposure to ozone alone in BALF. These observations also suggest clear EHC-93 particle-exposure specific differences in the lungs of exposed animals.

In this work, exposure to air pollutants affected the blood levels of hemoglobin variants, especially carboxyhemoglobin (COHb) and methemoglobin (Fig. 5). These changes were sustained even after 24 h post-exposure only in particle exposed animals. These hemoglobin variants are also known to form for instance during CO, and NO exposures [40–42] and have been associated with negative impacts on health [43]. We have reported previously an increase of heme oxygenase-1 gene expression in these same animals, notably in the lungs, heart and liver after 24 h recovery following inhalation of EHC-93 [37]. There are reports on particle exposure and heme oxygenase-1 levels in circulation [44]. This implies that deposition of ambient particles in the lungs can initiate a systemic response that activates endogenous CO production. It appears that in our model, sufficient CO was produced to shift COHb levels which can potentially decrease oxygen delivery to tissues. This is a significant observation that supports biological plausibility and adds to the weight of evidence for adverse cardiovascular impacts of respirable ambient particulate matter.

Although primary targets of inhaled pollutants are the lung lining and resident alveolar macrophages and primary local changes are anticipated here, we hypothesised that secondary effects probably can be seen in other tissues, such as plasma due to the onset of free radical cascades. Moreover, in the case of particle exposures, secondary effects in extra-pulmonary sites can also be due to translocation of particles to other sites. Our results indicated that plasma profiles of ROS products m-, o-, p-tyrosines were somewhat different from that of BALF profiles. Nevertheless, the profiles of 3-nitrotyrosine and the 3—nitrotyrosine to L-DOPA ratio in plasma exhibited similarity to those in BALF. Furthermore, o-tyrosine levels showed dose-related increases both at immediately post-exposure to ozone alone as well as to EHC-93 alone, and these changes were sustained even at 24 h post-exposure (Fig. 6). However, when exposed to O_3_ plus EHC-93 mixture, o-tyrosine levels exhibited significant inter-pollutant interactions (Ozone x EHC-93, *p* = 0.025). This can be reflective of a secondary oxidative stress condition set beyond what we noted at the target sites. The observation of relatively steeper slope for the plasma o-tyrosine dose—response curve with EHC-93 exposure compared to ozone suggests that in animals exposed to particles, metal-catalyzed oxidation reactions could favour o-tyrosine formation in plasma as well [34, 45, 46].

Even though inhalation of O_3_ led to some 3—nitrotyrosine formation in plasma, the effect was not dose-dependent. In contrast, inhalation exposure to EHC-93 led to a linear dose-related increase in plasma 3-nitrotyrosine levels. When animals were exposed to the pollutant mixtures, inter-pollutant interactions were noticed in the 3-nitrotyrosine levels. Yet at 24 h post-exposure to pollutant mixtures, this RNS marker appeared to be solely affected by EHC-93 particle exposure. Interestingly, although we did not see significant changes in the plasma levels of 8-isoPGF2α, a lipid oxidation marker (Additional file 2), it appeared to track the 3-nitrotyrosine levels in circulation. It is to be noted here that “superoxide anion” is implicated in the formation of both 8-isoPGF2α and 3-nitrotyrosine. Similar observations have been noted with diesel exposures [47].

Plasmatic levels of endothelin-1 (ET-1), a potent vasoconstrictor peptide and its precursor big endothelin-1 (BET-1) were increased in animals immediately post-exposure to O_3_ or EHC-93, but pollutant co-exposures led to a negative interaction. Endothelins are a family of vasoactive peptides (21 amino acids) consisting of three distinct isoforms, endothelin-1 (ET-1), endothelin-2 (ET-2) and endothelin-3 (ET-3) and are coded by different genes [48]. The mature endothelins (ET-1, −2 and −3) are produced *in vivo* from cleavage of their corresponding big endothelins (BET) by endothelin-converting enzymes [49, 50]. Endothelin-1, the most abundant isoform with diverse biological activity, has been implicated in several diseases, particularly in the progression of cardiovascular disease [51, 52]. At 24 h post-recovery, the effects due to individual pollutants were somewhat sustained for both BET-1 and its conversion product ET-1. This is consistent with our previous findings on animals and humans exposed to air pollutants [23, 27]. However, when exposed to the pollutant mixture, at 24 h post exposure, both BET-1 and ET-1 levels in circulation decreased compared to when exposed to individual pollutants. This decrease in BET-1 and ET-1 (ET_1–21_) levels after exposure to high dose pollutant mixtures at both immediately and 24 h post-exposure can be attributed to a heightened alternative MMP-2 directed BET-1 cleavage, leading to the formation of ET_1–32_ as discussed in our previous report [43]. The decrease in ET-1 levels in plasma could also be due to increased clearance via ET [B] receptor binding [53], at high dose exposure to pollutant mixtures. Although there were no significant changes noted with the levels of circulating ET-2 isoform, the ET-3 isoform tracked the profile changes of ET-1 through the different exposure conditions.

The plasma ET-1/BET-1 ratio values (Fig. 7e) can serve as a measure of endothelin converting enzyme (ECE) activity with air pollutant exposures. These results suggested that air pollutants individually or as mixtures perhaps impacted on ECE activity both immediately and at 24 h post-exposure. The plasmatic ET-1/ET-3 ratio profile (Fig. 7f) can function as a descriptor of balance between vasoconstrictive and vasorelaxing mechanisms [54], and our data suggest that at 24 h post-exposure to pollutant mixtures, compensatory vasodilatory mechanisms may be triggered to increase ET-1 clearance and/or decrease *de novo* synthesis. Our observations of a positive correlation between ET-1 and ET-3 in plasma, and that plasmatic ET-1 levels were predicted by plasma BET-1 levels argue in favour of co-regulation mechanisms [54]. Also, the plasma free nitrite analysis results (See Additional file 3) exhibited an increasing trend with particle exposures, but non-monotonic changes were seen with increasing ozone doses, immediately post-exposure (not significant), and systemic inflammation with increased iNOS activity cannot be ruled out [43]. However, at 24 h post-exposure, free nitrite levels in circulation were relatively higher (not significant) in animals exposed to the pollutant mixture consistent with enhanced compensatory effect as mentioned before.

Oxidative stress has been implicated in vascular dysfunction [55, 56]. In our work, Correlation analysis results exhibited significant (*p* < 0.05) positive association between BALF m-tyrosine levels and plasmatic BET-1 (*r* = 0.330) and ET-1 (*r* = 0.309) levels. Similarly, BALF o-tyrosine levels were positively correlated with and plasmatic BET-1 (*r* = 0.245), ET-1 (*r* = 0.352) and ET-3 (*r* = 0.255) levels. Our regression analysis results confirmed these findings as well. m-Tyrosine and o-tyrosine are OH• (ROS/RNS)-derived metabolites. Furthermore, we have observed that treatment of these animals with a superoxide dismutase mimetic AEOL 10150 abrogated pollutant induced increase in 3-nitrotyrosine and ET-1 levels.(to be published) These results together imply that air pollution exposure can trigger oxidative/nitrative stress in lungs that can impact on the endothelinergic system.

Interestingly, disruption of the endothelinergic system and formation of 3-nitrotyrosine are two well documented central mechanisms in many disease processes including atherosclerosis, pulmonary hypertension, asthma, neurological disorders, cancer and with air pollution and radiation exposure (ozone is known as radiomimetic gas) [57–67]. It is therefore feasible that air pollutants can potentially influence such pathologies through these mechanistic pathways. This is in line with reports on health-compromised individuals such as those with existing cardiovascular disease, asthma, diabetes and cancer as well as pregnant women, the elderly and children being more susceptible to air pollution [68–72].

Our findings suggest that exposure to ozone and ambient particles can lead to systemic nitrative stress, activation of the endothelinergic system, and elevate endogenous CO production with a potential for decreasing oxygen delivery. Our observations argue for biological plausibility and add to the weight of evidence for adverse health impacts of ambient air pollutants thus contributing to the advancement of risk assessment approaches. The kinetics of interaction of oxidative and nitrative pathways in our model offer additional insight into the mechanistic bases of epidemiological findings on the vulnerability of selected population to air pollution.

## Conclusions

Exposures to individual air pollutants result in pollutant-specific oxidative/nitrative stress changes in lungs and in circulation. Meanwhile, pollutant mixtures can either cause additive or suppressive effects or reflect effects due to a single component depending on which endpoint is being looked at. In healthy rats, compensatory mechanisms can be activated. Our results indicate that air pollutant exposures can initiate primary and secondary systemic oxidative or nitrative stress, changes in hemoglobin variants notably COHb levels and endothelinergic system that could modulate different pathologies, if compensatory mechanisms are not viable. Future studies using high-content proteomic analyses to investigate protein expression levels along with functional modifications can validate and provide further insight into these air pollutant exposure-specific mechanistic pathways relevant to diseases through a systems biology approach.

## Methods

### Chemicals

Dulbecco’s phosphate-buffered saline (PBS, calcium and magnesium-free), ethylenediaminetetraacetic acid (EDTA), diethylenetriaminepentaacetic acid (DETPA), phenylmethylsulfonyl fluoride (PMSF), trifluoroacetic acid (TFA), 3,4-dichloroisocoumarin sodium acetate, trisodium salt of citric acid, octanesulfonic acid sodium salt (OSA), trizma hydrochloride, trizma base, NADPH, sodium hydroxide, nitrate reductase, sodium nitrite, potassium nitrate, molecular weight cut-off filters (10 and 30 kDa) and standards of p-tyrosine, m-tyrosine, o-tyrosine, 3-nitrotyrosine, L-3,4-dihydroxyphenylalanine (L-DOPA), 2,4 dinitrophenylhydrazine (2,4-DNPH), 1,1,3,3-tetra-methoxy-propane, and endothelin isoform standards (ET-1, ET-2, ET-3) were purchased from Sigma (St. Louis, MO, USA). Analytical reagent-grade NaCl, and HCl were from BDH (Toronto, Canada). Reagent-grade acetone, acetonitrile, and methanol were from commercial suppliers. Butylated hydroxytoluene (BHT) was from United States Biochemical Corporation (Cleveland, OH, USA). Carbonyl-DNPH mix 1 standard was purchased from Supelco (Bellefonte, PA). Big ET-1 standard was from Bachem Americas, Inc. (Torrence, CA, USA). Deionized water (DI water) was from a super-Q plus high purity water system (Millipore, Bedford, MA, USA). UHP-grade compressed nitrogen was supplied by Matheson Gas products (Whitby, Canada). Amber glass vials and screw caps with septa were purchased from Chromatographic specialities, Inc. (Brockville, ON). 2, 3-diaminonaphthalene was obtained from Molecular Probes (Eugene, OR). Enzyme immunoassay (EIA) kit for 8-isoPGF-2α analysis was purchased from Cayman chemicals (Ann Arbor, Michigan, USA).

### Animals

Specific pathogen-free healthy Fischer-344 male rats (200–250 g) were obtained from Charles River (St. Constant, Quebec, Canada). The animals were housed in individual plexiglass cages on wood-chip bedding under HEPA filtered air and held to a 12 h dark/light cycle. Food and water were provided ad libitum. All experimental protocols were reviewed and approved by the Animal Care Committee of Health Canada. Animals were randomly allocated to the different exposure groups (Group size: *N* = 8/air pollutant exposure group; *N* = 17/air control group).

### Inhalation exposures

Fischer 344 rats were trained in nose-only exposure tubes over five consecutive days and were exposed for 4 h within an interaction matrix of ozone (0, 0.4, 0.8 ppm) factored by EHC-93 particle (0, 5, 50 mg/m^3^) concentrations by nose-only inhalation, essentially as described before [15, 24, 27]. Animals were euthanized immediately after exposure (0 h recovery for the complete 0, 0.4, 0.8 ppm O_3_ × 0, 5, 50 mg/m^3^ EHC-93 particle matrix) and 24 h post-exposure recovery in filtered air (24 h recovery for the 0, 0.8 ppm O_3_ × 0, 50 mg/m^3^ EHC-93 particle matrix). Note that exposure sessions in the particle-dedicated nose-only exposure system to different particle concentrations had to be conducted on different days. In order to minimize day-of-exposure biases, exposures were staggered and air control animals were included in a parallel clean-air nose-only exposure system. For this reason, while treatment groups (any exposure containing ozone and/or particles) were blocked to avoid biases and are balanced, the number of matched animals in the air control groups (*N* = 17) are higher than for the treatment groups (*N* = 8–14), resulting in an overall unbalanced design. In short, the experimental design is a three-factor design for the high dose combinations with OZONE (levels 0, 0.8 ppm O3) and EHC-93 particles (levels 0, 50 mg/m^3^).

Briefly, ozone for the experimental atmosphere was generated from oxygen in a silent arc generator (Model 200, Sanders, Uetze, Germany), sampled at the inhalation ports through a Teflon filter and was monitored by UV spectrometry (Model 1003-AH, Dasibi Environmental, Glendale, CA). Ozone concentrations were stabilized by feedback control and varied ± 5 % of the target concentration. The EHC-93 particles were dispersed in a venturi (Powder Disperser Model 3433, TSI St. Paul, MN) and directed to a flow-past, nose-only exposure inhalation manifold (CH technologies, Westwood, NJ). One or two stacks of inhalation manifolds were used for a flow rate of 12–24 L/min (1 L/min per port). The EHC-93 particle concentrations were tested at the ports by isokinetic sampling on 0.2 μm Teflon filters (Gelman Sciences, Ann Arbor, MI). Real-time measurements of particle size and counts were done by isokinetic sampling at the inhalation ports (Lasair model 301, Particle Measuring Systems, Boulder, CO). Count median diameter (CMD) and the mass median aerodynamic diameter (MMAD) were obtained by laser optic counting and cascade impactor analyses (seven-stage Mercer type, Intox, Albuquerque, NM), respectively. Resuspended EHC-93 particles contain two respirable modes at 1.3 μm aerodynamic diameter (D_AE_) (mode 1, 20 % of aerosol mass) and 3.6 μm D_AE_ (mode 2, 35 % of aerosol mass), and an additional coarse mode at 15 μm D_AE_ (mode 3), which account for the remaining 45 % of the aerosol mass [24].

### Biological samples

Animals were anaesthetized with sodium pentobarbital (65 mg/kg, i.p.). The trachea was exposed and cannulated, blood was withdrawn from the abdominal aorta using heparinized syringes and transferred into vacutainer tubes containing the sodium salt of ethylenediaminetetraacetic acid (10 mg/mL) and phenyl methyl sulfonyl fluoride (1.7 mg/mL), mixed gently, and placed on ice. The diaphragm was then punctured, the lungs were filled by intra-tracheal instillation of warm saline (37 °C) at 30 mL/kg body weight [73, 74]. Lungs were massaged gently by rubbing the thoracic cage. Saline was aspirated and re-injected, twice, and the primary bronchoalveolar lavage fluid (BALF) was collected in a cold centrifuge tube. Secondary BALF was obtained with additional volumes of saline (5 mL/rat), three times, to increase the yield of lavage cells. The primary and secondary lavage fluids were centrifuged (300 × g for 10 min at 4 °C) separately to isolate cells from the supernatants. Primary BALF supernatants were used to analyse biochemical endpoints. Secondary BALF supernatants were discarded. Cell pellets from both primary and secondary BALF supernatants were combined to recover the total BAL cells.

### Analyses of bronchoalveolar lavage

#### BAL cytology

Lung bronchoalveolar lavage cells were counted using a Coulter Multisizer II (Coulter Canada, Ville St-Laurent, Que., Canada), and differential cell counts were obtained from cytospin preparations using Wright stain and with use of Ames-Haematic slide stainer following standard procedures [75].

#### Lipid oxidation markers in BAL cells

One subset of aliquots of total BAL cells (from the ozone (0, 0.8 ppm) and particle (0, 50 mg/m^3^) exposures immediately post recovery) of known cell counts were treated with stabilizers (25 μL of aqueous 0.1 M DETPA solution and 25 μL of 0.3 M BHT solution in isopropanol). These samples were processed for lipid oxidation products (e.g. aldehydes) analysis, following the procedure reported by Kumarathasan et al. [16]. Briefly, already stabilized total BAL cells were lysed by sonication (15 min), and cell suspensions were derivatized with a saturated solution of 2,4-DNPH in 2 N HCl at room temperature for 1 h. The DNPH derivatives of the aldehydes were extracted with toluene, dried over anhydrous sodium sulphate/silanized glass-wool plug, concentrated under N_2_ and were analyzed by a previously reported GC-MS method by Kumarathasan et al. [16] and the analyte peak intensities were recorded.

#### BALF pH and total protein

pH values of the primary BALF supernatants were initially recorded using pH strips. Protein concentrations in BALF supernatants were measured after appropriate dilution in deionized water by Coomassie brilliant blue dye binding assay [76]. The remainder of the BALF supernatants were vortexed with 50 μL of aqueous 0.1 M DETPA solution and 50 μL of 0.3 M BHT solution in isopropanol to prevent autoxidation of samples. Aliquots of the stabilized BALF supernatants were analysed for protein carbonyls, protein oxidation and nitration markers.

#### BALF protein carbonyls

Aliquots (2 mL) of stabilized BALF supernatant samples were initially concentrated under a flow of N_2_ to 250 μL. These samples were then treated with 10 % ice-cold TCA, vortexed and centrifuged at 9019 × g for 10 min. Supernatants were discarded and the precipitates were treated with 250 μL aliquots of 2,4-DNPH in 2 N HCl, homogenized, vortexed and allowed to react at room temperature for 1 h. DNPH-derivatized samples were then centrifuged at 9019 × g for 10 min, and the precipitate was washed twice with a 1:1 mixture (by volume) of ethylacetate:ethanol. Washed precipitates were dissolved in 500 μL of 6 M guanidine HCl solution (pH = 1.8). These samples were analysed at 280 and 363 nm by UV/Visible spectrometry (MiltonRoy Spectrometer, LABEQUIP LTD, Markham, ON, Canada).

#### Markers of protein oxidation and nitration

Markers of both reactive oxygen and nitrogen species generation were analyzed using the HPLC-Coularray method described by [77]. In brief, 750 μL aliquots of BALF supernatant samples stabilized with DETPA and BHT were evaporated to 250 μL under a flow of N_2_. These samples were de-proteinized by use of acidified acetone, evaporated to concentrate and were subsequently clarified using molecular weight cut-off filters (30 kDa). Nitrogen-dried samples were then reconstituted with 100 μL of acidified water prior to analysis by a HPLC-Coularray method. Initial separation of analytes was carried out on a LC-18 reversed phase column (25 cm length, 4.6 mm id, 5 μm particle size; Supelco, Oakville, ON) by isocratic elution using a citrate-acetate buffer (pH = 4.7) mobile phase containing OSA as the ion-pair reagent. Separated analytes were measured by coulometric array detection using a set of eight electrodes at different applied potentials.

### Analyses of blood and plasma

#### Sample preparation

Whole blood samples were initially aliquoted for free nitrite analysis and for the analysis of haemoglobin (Hb) variants. Remainder of the whole blood sample was clarified to obtain plasma following the procedure described by Kumarathasan et al. [36]. Briefly, blood samples were centrifuged at 1448 × g for 10 min to obtain plasma. Plasma samples were recovered and vortexed with 50 μL of aqueous 0.1 M DETPA solution and 50 μL of 0.3 M BHT solution in isopropanol to prevent any post-mortem changes due to autoxidation. Two sets of aliquots (250 μL) of plasma samples were used for 8-isoPGF2α and for protein oxidation, nitration marker analyses, while another set of aliquots was used for analysis of circulating levels of vaso-regulatory peptides, endothelins.

#### Hemoglobin variants in blood

Aliquots (50 μL) of stabilized whole blood samples were diluted with deionized water to a final volume of 1 mL, were analyzed at 500, 569, 577 and 620 nm [78, 79] for methemoglobin, carboxyhemoglobin, oxyhemoglobin and sulfhemoglobin respectively by UV/Visible spectrometry (MiltonRoy Spectrometer, LABEQUIP LTD, Markham, ON, Canada) and the absorbance values were recorded. Carboxyhemoglobin and oxyhemoglobin absorbances were corrected for interferences.

#### Plasma 8-isoPGF2α

Aliquots of plasma (250 μL) samples stabilized with DETPA and BHT were used for the 8-isoPGF2α analysis. Antibody-based affinity purification of plasma samples was conducted following the procedure described by Bielecki et al. [80]. Purified plasma samples were then analyzed by a competitive enzyme immunoassay (EIA) for 8-isoPGF2α using the EIA kit from Cayman Chemicals (Ann Arbor, Michigan, USA).

#### Protein oxidation and nitration markers in plasma

Oxidative stress levels in rat plasma were measured by analysis of protein oxidation and nitration products using the HPLC-Coularray method described by Kumarathasan and Vincent [77]. Here, 250 μL aliquots of plasma samples stabilized with DETPA and BHT were deproteinized using acidified acetone, evaporated to concentrate, clarified using molecular weight cut-off filters (30 kDa), dried under N_2_ flow and were reconstituted with 100 μL of acidified water prior to analysis by the HPLC-Coularray method as described above for the oxidative stress marker levels in BALF supernatants.

#### Circulating endothelin isoforms

This procedure was conducted as described by Kumarathasan et al. [36]. Briefly, aliquots of plasma samples (250 μL) were treated with 3,4-dichloroisocoumarin solution in isopropanol to prevent conversion of big ET-1 (BET-1) to ET-1 during sample processing. These samples were then deproteinized by acidified acetone, evaporated to concentrate and were cleaned up using molecular weight cut-off filters (30 kDa). Clarified samples were dried under a N_2_ flow and were reconstituted in phosphate buffered saline and were analyzed by a reversed phase HPLC-Fluorescence system. Initial separation of endothelin isoforms were carried out on a LC-318 column (25 cm length, 4.6 mm id, 5 μm particle size; Supelco, Oakville, ON) by gradient elution using water-acetonitrile mobile phase [A-30 % acetonitrile (aq); B-90 % acetonitrile (aq)] with 0.19 % of TFA used as the ion-pair reagent. Analytes were measured by fluorescence detection at excitation and emission wavelengths of 240 and 380 nm, respectively.

#### Plasma nitrite

Plasma nitrite analysis was done to determine free nitrite levels as a supporting endpoint. These analyses were conducted on the immediately post-exposure samples due to the availability of sample volumes. This analysis method is based on the thermolysis of all NO-related compounds *in vivo* such as nitrosyl metal complexes, nitrite/nitrate, S-nitroso compounds in plasma to nitrate, followed by enzymatic reduction to nitrite that was derivatized using a fluorescent probe for detection by fluorescence. Nitrite analysis was conducted following a previously reported procedure [67]. In essence, plasma samples (50 μL) treated with DETPA and BHT were thermolyzed at 86 °C, cooled and filtered via 10 kDa molecular weight cut-off filters. These samples were treated with nitrate reductase from Sigma Chemical (St. Louis, MO) at room temperature for an hour, and were derivatized with 2,3 diaminonaphthalene from Molecular Probes (Eugene, OR). Fluorescence measurements were made at Ex λ = 360 nm; Em λ = 460 nm on a Cytofluor 2350 multiplate fluorescence detector (Millipore, Bedford, MA).

### Relevance of pollutant dose-regimen used in this work to environmental exposures

To estimate a reference particle deposition in human lungs in a realistic environmental exposure scenario, we used an average tidal volume of 875 mL, average breathing frequency of 16 min^−1^ over the entire day (20.2 m^3^ air inhaled/d), oro-nasal breathing, and an alveolar surface area of 54 m^2^. For the 0.05–10 μm D_AE_ size range of urban particulate matter with size cut-off of 10 μm D_AE_ (PM_10_) consisting of a nucleation mode at 0.05 μm D_AE_ (5 % of mass), condensation mode at 0.2 μm D_AE_ (25 % of mass) and coarse mode at 5 μm D_AE_ (70 % of mass) deposition rates were considered at 0.20 for all three modes. Using these parameters and assuming a 24 h exposure to an average PM_10_ concentration of 175 μg/m^3^, the 95th percentile PM concentration in Toronto, would result into a reference total dose in the pulmonary compartment of humans in the order of 707 μg (175 μg/m^3^ × 20.2 m^3^ × 0.20), translating into an average surface-specific internal dose of 1.3 ng/cm^2^ alveolar surface area. Similarly, the peak centriacinar dose of ozone in human lungs can be considered as 30 × 10^−6^ μg O_3_/cm^2^/h per μg ambient O_3_/m^3^ [81]. Thus, for an 0.12 ppm ozone (236 μg O_3_/m^3^) exposure over 12 h (85 ng O_3_/cm^2^) followed by 0.06 ppm ozone for 12 h (42 ng O_3_/cm^2^), the total daily centriacinar peak surface-specific internal dose is estimated at 127 ng O_3_/cm^2^ [12].

For rats in our experiments, model assumptions were a tidal volume of 2.1 mL, breathing frequency of 102 min^−1^ (51.41 air inhaled /4 h exposure), strict nasal breathing, and an alveolar surface area of 0.34 m^2^. We have estimated deposition rates in the rat lung using the Multiple Path Particle Deposition software (MPPDep v1.11, RIVM Publications, Bilthoven, The Netherlands) as at 0.081 for the 1.3 μm D_AE_ mode (20 % of aerosol mass), 0.047 for the 3.6 μm D_AE_ (35 % of aerosol mass) and 0.000 for the 15 μm D_AE_ (45 % of aerosol mass). Therefore, the deposition of EHC-93 particles in the pulmonary compartment of the rats was estimated at 8.4 μg per 5 mg/m^3^ exposure concentration (5 μg/L × 51.41 × {[0.20 × 0.081] + [0.35 × 0.047] + [0.45 × 0.000]}) or 2.5 ng/cm^2^ alveolar surface area per 5 mg/m^3^ exposure concentration. The peak centriacinar dose of ozone in rat lungs can be estimated as 68 × 10^−6^ μg O_3_/cm^2^/h per μg ambient O_3_/m^3^ [81]. Thus, exposure of our rats to 0.4 ppm ozone (785 μg O_3_/m^3^) or 0.8 ppm ozone (1570 μg O_3_/ m^3^) over 4 h should have resulted in total centriacinar peak doses of 214 ng O_3_/cm^2^ and 427 ng O_3_/cm^2^, respectively.

It is not possible to conduct nose-only inhalation exposures of experimental animals over a 24 h period at a dose-rate that reproduces the human exposure experience. Nose-only exposures with animal restraint should not exceed 4 h, and thus the dose-rate must be 5–10 times that of the human exposure context to create an internal experimental dose in animals that approaches the surface area-specific internal dose in human. The ratios of EHC-93 doses within the respiratory compartment of the rats exposed for 4 h to 5 mg/m^3^ (2.5 ng/cm^2^) and 50 mg/m^3^ (25 ng/cm^2^) vs particle dose in human lungs during plausible 24 h exposure scenarios (1.3 ng/cm^2^) were within one order of magnitude (2 and 20-fold respectively). The ratios of ozone centriacinar peak doses in rats at 0.4 ppm ozone (214 ng O_3_/cm^2^) and 0.8 ppm ozone (427 ng O_3_/cm^2^) to the estimated internal dose for human during plausible environmental exposure scenario (127 ng O_3_/cm^2^) were within an order of magnitude (1.7 and 3.4-fold respectively). Nose-only exposures were kept to a minimum duration for ethical reasons and thus the dose rate in our study was relatively higher as opposed to an environmental exposure spread over 24 h period. Therefore, from the viewpoint of toxicological evaluation, the pulmonary deposition or experimental internal dose in our experimental animals are directly relevant to human health risk assessment, particularly if the following uncertainty factors are considered: interspecies differences in sensitivity to air pollutants (humans being more responsive than rats), increased sensitivity of the vulnerable population (humans with congestive heart failure or atherosclerosis more responsive than healthy individuals), and the possible decay of EHC-93 particle potency with collection by comparison to freshly functionalized particles instantaneously inhaled during real-time human exposures, albeit to extreme conditions. If a combined uncertainty of 100-fold is accepted from those factors, then the internal doses of particles and ozone created in our animal model become directly comparable to a human daily internal pulmonary dose (surface area- and compartment-specific) under more common exposure conditions, for example 24 h inhalation of 10 ppb ozone and 50 ug/m^3^ PM_10_.

### Statistical analyses

Results are expressed as mean ± standard error of the mean. Most of the endpoints are reported as fold-effect normalized to controls to remove day-day variation or animal batch-batch variation as mentioned before. Also, since multiple endpoints were analysed in this study, sample sizes varied per analysis (refer to Figure legends) based on available sample volume or sample loss.

The overall effects of air pollutant exposures were tested for statistical significance by three-way ANOVA with OZONE (levels 0, 0.8 ppm O_3_), EHC-93 (levels 0, 50 mg/m^3^ EHC-93) and RECOVERY (levels 0, 24 h post-exposure) as factors. The detailed dose-interaction model was then tested for statistical significance by two-way ANOVA with OZONE (0, 0.4, 0.8 ppm O3) and EHC-93 (0, 5, 50 mg/m^3^ EHC-93) as factors. For lipid oxidation product analyses in BAL cells, a two-way ANOVA analysis was performed with OZONE (levels 0, 0.8 ppm O3) and EHC-93 (0, 50 mg/m^3^ EHC-93) as factors. Tukey’s multiple pair-wise comparison procedure was applied to elucidate the patterns of significant effects (α =0.05). Data sets not meeting the assumptions of normality and equal variance for ANOVA were subjected to natural log (ln), square root or rank prior to the analyses. Spearman Rank Order and Pearson Product Moment correlation analyses were done to test associations between different endpoints in different biological compartments. Backward stepwise regression analysis was also conducted to determine early markers predictive of late biological events such as changes in endothelinergic system. For the purpose of clarity we have reported significant factor interactions in the Results section. Significant main effects are described in text only if they were not part of a significant factor interaction. Statistical significance reported in the figure legends and Additional files 4, 5, 6 and 7 refer to the Tukey’s post-hoc comparison as directed by significant main effects or factor interactions from the ANOVA tests. All statistical analyses were conducted using Sigma Stat software (Version 3.5, Chicago, IL).

## Additional files

Additional file 1:
**Protein carbonyl contents in BALF supernatant (mean ± SEM) (Sample size,** ***n*** **= 7–8/air pollutant exposure group;** ***n*** **= 11/air control group).** (DOCX 23 kb) Additional file 2:
**Modulation of the lipid oxidation marker** **8-isoPGF2α** **in plasma (mean ± SEM). (Sample size,** ***n*** **= 3/air pollutant exposure group;** ***n*** **= 6/air control group).** (DOCX 22 kb) Additional file 3:
**Plasma nitrite profiles (mean ± SEM) (Sample size,** ***n*** **= 3/air pollutant exposure group;** *n* **= 6/air control group).** (DOCX 22 kb) Additional file 4:
**BAL Cells and Fluid.** 3-Way ANOVA with EHC-93 (0, 50 mg/m3), Ozone (0, 0.8 ppm) and Recovery (0 h, 24 h) as factors. (DOCX 14.6 kb) Additional file 5:
**Blood and Plasma.** 3-Way ANOVA with EHC-93 (0, 50 mg/m^3^), Ozone (0, 0.8 ppm) and Recovery (0 h, 24 h) as factors. (DOCX 15 kb) Additional file 6:
**BAL Cells and Fluid.** 2-Way ANOVA with EHC-93 (0, 5, 50 mg/m3) and Ozone (0, 0.4, 0.8 ppm) as factors immediately after exposure. (DOCX 14.8 kb) Additional file 7:
**Blood and Plasma.** 2-Way ANOVA with EHC-93 (0, 5, 50 mg/m^3^) and Ozone (0, 0.4, 0.8 ppm) as factors immediately after exposure. (DOCX 16 kb)

## Abbreviations

BALF: Bronchoalveolar lavage fluid
ROS: Reactive oxygen species
RNS: Reactive nitrogen species
BET-1: Big endothelin-1
ET-1: Endothelin-1
TNFα: Tumor necrosis factor alpha
