# Supplementary figures and images for: Nitrative stress, oxidative stress and plasma endothelin levels after inhalation of particulate matter and ozone
Source: Part Fibre Toxicol. 2015 Sep 17;12:28. doi: 10.1186/s12989-015-0103-7 (PMC4573945; doi:10.1186/s12989-015-0103-7)

Additional File 1. BALF Protein Carbonyl
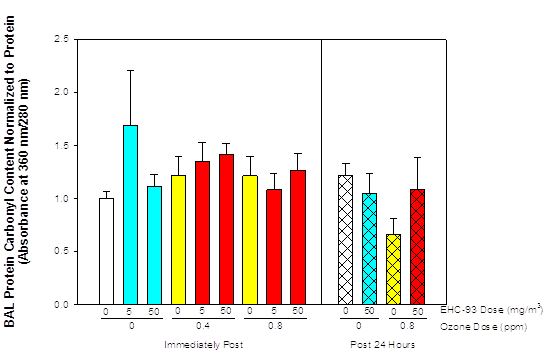

Supplement: Additional file 1: — Protein carbonyl contents in BALF supernatant (mean ± SEM) (Sample size, n = 7–8/air pollutant exposure group; n = 11/air control group). (DOCX 23 kb) [file 12989_2015_103_MOESM1_ESM.docx]

Additional File 2. Plasma Isoprostane.


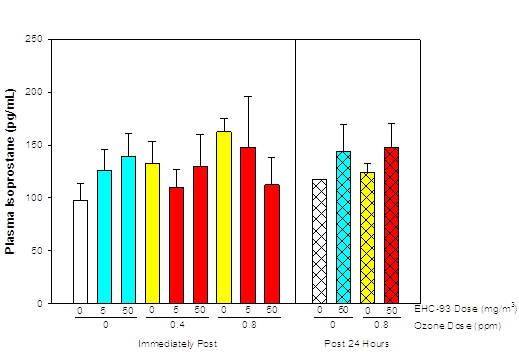

Supplement: Additional file 2: — Modulation of the lipid oxidation marker 8-isoPGF2α in plasma (mean ± SEM). (Sample size, n = 3/air pollutant exposure group; n = 6/air control group). (DOCX 22 kb) [file 12989_2015_103_MOESM2_ESM.docx]

Additional File 3. Plasma Nitrite.


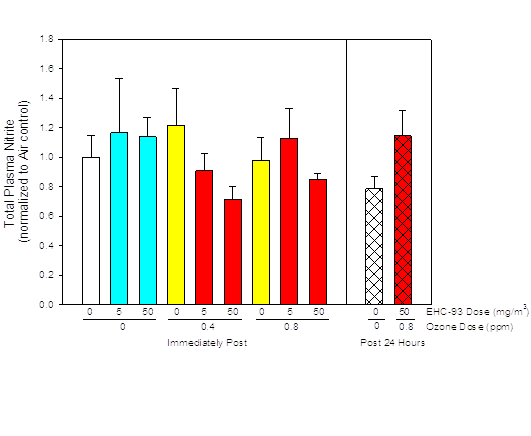

Supplement: Additional file 3: — Plasma nitrite profiles (mean ± SEM) (Sample size, n = 3/air pollutant exposure group; n = 6/air control group). (DOCX 22 kb) [file 12989_2015_103_MOESM3_ESM.docx]
