# Supplementary material for: Nitrative stress, oxidative stress and plasma endothelin levels after inhalation of particulate matter and ozone
Source: Part Fibre Toxicol. 2015 Sep 17;12:28. doi: 10.1186/s12989-015-0103-7 (PMC4573945; doi:10.1186/s12989-015-0103-7)
Supplement: Additional file 4: — BAL Cells and Fluid. 3-Way ANOVA with EHC-93 (0, 50 mg/m3), Ozone (0, 0.8 ppm) and Recovery (0 h, 24 h) as factors. (DOCX 14.6 kb) [file 12989_2015_103_MOESM4_ESM.docx]

Additional File 4. Table: BAL Cells and Fluid. 3-Way ANOVA with EHC-93 (0, 50 mg/m3), Ozone (0, 0.8 ppm) and Recovery (0 h, 24 h) as factors.

| **Endpoint** | **Significant Effects** | **P** | **Tukey (p<0.05)** |
| --- | --- | --- | --- |
| Total Cell Count | Ozone x Recovery | p<0.001 | 0 vs 0.8 ppm O_3_ within 24h Recovery  0h vs 24h Recovery within 0 ppm O_3_  0h vs 24h Recovery within 0.8 ppm O_3_ |
| Total Macrophages | Ozone x Recovery | p<0.001 | 0 vs 0.8 ppm O_3_ within 24h Recovery |
| Total Neutrophils | Ozone x Recovery | p=0.011 | 0 vs 0.8 ppm O_3_ within 24h Recovery  0h vs 24h within 0.8 ppm O_3_ |
|  | Ozone x EHC-93 | p=0.030 |  |
| Mature Macrophages | Ozone x Recovery | p=0.029 | 0 vs 0.8 ppm O_3_ within 24h Recovery |
| BALF pH | Ozone x Recovery | p<0.001 | 0h vs 24h Recovery within 0 ppm O_3_  0h vs 24h Recovery within 0.8 ppm O_3_ |
|  | EHC-93 | p=0.048 | 0 vs 50 mg/m_3_ EHC-93 |
| BALF Protein | Ozone x Recovery | p<0.001 | 0h vs 24h within 0 ppm O_3_  0h vs 24h 2ithin 0.8 ppm O_3_ |
| BALF o-Tyrosine | Recovery | p<0.050 | 0h vs 24h |
| BALF m-Tyrosine | Ozone x Recovery | p<0.001 | 0 vs 0.8 ppm O_3_ within 24h Recovery |
| BALF p-Tyrosine | Ozone | p<0.001 | 0 vs 0.8 ppm O_3_ |
| BALF 3-Nitrotyrosine | Ozone x EHC-93 | p=0.011 | 0 vs 0.8 ppm O_3_ within 0 mg/m^3^ EHC-93  0 vs 0.8 ppm O_3_ within 50 mg/m^3^ EHC-93  0 vs 50 mg/m^3^ EHC-93 within 0 ppm O_3_  0 vs 50 mg/m^3^ EHC-93 within 0.8 ppm O_3_ |
|  | Recovery | p=0.013 | 0h vs 24h Recovery |
| BALF 3‑Nitrotyrosine/L‑DOPA | EHC-93 | p<0.001 | 0 vs 50 mg/m^3^ EHC-93 |
