# Supplementary material for: Nitrative stress, oxidative stress and plasma endothelin levels after inhalation of particulate matter and ozone
Source: Part Fibre Toxicol. 2015 Sep 17;12:28. doi: 10.1186/s12989-015-0103-7 (PMC4573945; doi:10.1186/s12989-015-0103-7)
Supplement: Additional file 5: — Blood and Plasma. 3-Way ANOVA with EHC-93 (0, 50 mg/m3), Ozone (0, 0.8 ppm) and Recovery (0 h, 24 h) as factors. (DOCX 15 kb) [file 12989_2015_103_MOESM5_ESM.docx]

Additional File 5. Table: Blood and Plasma. 3-Way ANOVA with EHC-93 (0, 50 mg/m^3^), Ozone (0, 0.8 ppm) and Recovery (0 h, 24 h) as factors.

| **Endpoint** | **Significant Effects** | **P** | **Tukey (p<0.05)** |
| --- | --- | --- | --- |
| Methemoglobin | EHC-93 x Ozone | p=0.024 | 0 vs 0.8 ppm O_3_ within 0 mg/m^3^ EHC-93 |
|  | EHC-93 x Recovery | p=0.035 | 0h vs 24h Recovery within 50 mg/m^3^ EHC-93 |
| Sulfhemoglobin | NS |  |  |
| Carboxyhemoglobin | EHC-93 | p<0.001 | 0 vs 50 mg/m^3^ EHC-93 |
|  | Recovery | p=0.044 | 0h vs 24h |
| Oxyhemoglobin | NS |  |  |
| Plasma m-Tyrosine | NS |  |  |
| Plasma o-Tyrosine | Ozone x EHC-93 | p=0.025 | 0 vs 0.8 ppm O_3_ within 0 mg/m^3^ EHC-93  0 vs 50 mg/m^3^ within 0 ppm O_3_ |
| Plasma p-Tyrosine | Ozone x Recovery | p<0.001 | 0 vs 0.8 ppm O_3_ within 0h Recovery  0 vs 0.8 ppm O_3_ within 24h Recovery |
|  | EHC-93 main effect | p<0.001 | 0 vs 50 mg/m^3^ EHC-93 |
| Plasma 3-Nitrotyrosine | Ozone x EHC-93 | p=0.026 | 0 vs 0.8 ppm O_3_ within 0 mg/m^3^ EHC-93  0 vs 50 mg/m^3^ EHC-93 within 0 ppm O_3_ |
| Plasma 3‑Nitrotyrosine/L‑DOPA | EHC-93 | p=0.044 | 0 vs 50 mg/m^3^ EHC-93 |
| Plasma BET-1 | Ozone x EHC-93 | p<0.001 | 0 vs 0.8 ppm O_3_ within 0 mg/m^3^ EHC-93  0 vs 0.8 ppm O_3_ within 50 mg/m^3^ EHC-93  0 vs 50 mg/m^3^ within 0 ppm O_3_  0 vs 50 mg/m^3^ within 0.8 ppm O_3_ |
| Plasma ET-1 | Ozone x EHC-93 | p<0.001 | 0 vs 0.8 ppm O_3_ within 0 mg/m^3^ EHC-93  0 vs 0.8 ppm O_3_ within 50 mg/m^3^ EHC-93  0 vs 50 mg/m^3^ within 0 ppm O_3_  0 vs 50 mg/m^3^ within 0.8 ppm O_3_ |
| Plasma ET-2 | NS |  |  |
| Plasma ET-3 | Ozone x EHC-93 | p=0.021 | 0 vs 0.8 ppm O_3_ within 0 mg/m^3^ EHC-93 |
| Ratio ET-1/BET-1 | Ozone x Recovery | p=0.041 | 0h vs 24h Recovery within 50 mg/m^3^ EHC-93  0 vs 50 mg/m^3^ EHC-93 within 24h Recovery |
|  | Ozone x EHC-93 | p<0.001 | 0 vs 0.8 ppm O_3_ within 0 mg/m^3^ EHC-93  0 vs 50 mg/m^3^ within 0 ppm O_3_ |
| Ratio ET-1/ET-3 | Ozone x EHC-93 | p<0.001 | 0 vs 0.8 ppm O_3_ within 0 mg/m^3^ EHC-93  0 vs 0.8 ppm O_3_ within 50 mg/m^3^ EHC-93  0 vs 50 mg/m^3^ within 0 ppm O_3_  0 vs 50 mg/m^3^ within 0.8 ppm O_3_ |
