# Supplementary material for: Nitrative stress, oxidative stress and plasma endothelin levels after inhalation of particulate matter and ozone
Source: Part Fibre Toxicol. 2015 Sep 17;12:28. doi: 10.1186/s12989-015-0103-7 (PMC4573945; doi:10.1186/s12989-015-0103-7)
Supplement: Additional file 6: — BAL Cells and Fluid. 2-Way ANOVA with EHC-93 (0, 5, 50 mg/m3) and Ozone (0, 0.4, 0.8 ppm) as factors immediately after exposure. (DOCX 14.8 kb) [file 12989_2015_103_MOESM6_ESM.docx]

Additional File 6. Table: BAL Cells and Fluid. 2-Way ANOVA with EHC-93 (0, 5, 50 mg/m3) and Ozone (0, 0.4, 0.8 ppm) as factors immediately after exposure.

| **Endpoint** | **Significant Effects** | **P** | **Tukey (p<0.05)** |
| --- | --- | --- | --- |
| Total Cell Count | Ozone | p<0.001 | 0 vs 0.8 ppm O_3_  0.4 vs 0.8 ppm O_3_ |
| Total Macrophages | Ozone x EHC-93 | p=0.024 | 0 vs 0.8 ppm O_3_ within 0 mg/m^3^ EHC-93  0.4 vs 0.8 ppm O_3_ within 0 mg/m^3^ EHC-93  0 vs 0.4 ppm O_3_ within 5 mg/m^3^ EHC-93  0 vs 0.8 ppm O_3_ within 5 mg/m^3^  0 vs 0.8 ppm O_3_ within 50 mg/m^3^  0 vs 5 mg/m^3^ within 0 ppm O_3_ |
| Total Neutrophils | NS |  |  |
| Mature Macrophages | NS |  |  |
| Cell Formaldehyde | Ozone | p=0.007 | 0 vs 0.8 ppm O_3_ |
| Cell Propanal | Ozone | p=0.010 | 0 vs 0.8 ppm O_3_ |
| Cell 4-Hydroxynonenal | Ozone | p=0.008 | 0 vs 0.8 ppm O_3_ |
| BALF pH | Ozone | p<0.001 | 0 vs 0.8 ppm O_3_  0.4 vs 0.8 ppm O_3_ |
| BALF Protein | Ozone | p<0.001 | 0 vs 0.8 ppm O_3_  0.4 vs 0.8 ppm O_3_ |
| BALF o-Tyrosine | Ozone | p<0.001 | 0 vs 0.4 ppm O_3_  0.4 vs 0.8 ppm O_3_ |
| BALF m-Tyrosine | Ozone | p<0.001 | 0 vs 0.8 ppm O_3_  0.4 vs 0.8 ppm O_3_ |
| BALF p-Tyrosine | Ozone | p<0.001 | 0 vs 0.4 ppm O_3_  0 vs 0.8 ppm O_3_ |
| BALF 3-Nitrotyrosine | EHC-93 | p=0.011 | 0 vs 50 mg/m^3^ EHC-93  5 vs 50 mg/m^3^ EHC-93 |
| BALF 3‑Nitrotyrosine/L‑DOPA | Ozone x EHC-93 | p=0.032 | 0 vs 50 mg/m^3^ EHC-93 within 0 ppm O_3_  5 vs 50 mg/m^3^ EHC-93 within 0 ppm O_3_  0 vs 50 mg/m^3^ EHC-93 within 0.8 ppm O_3_ |
