# Supplementary material for: Nitrative stress, oxidative stress and plasma endothelin levels after inhalation of particulate matter and ozone
Source: Part Fibre Toxicol. 2015 Sep 17;12:28. doi: 10.1186/s12989-015-0103-7 (PMC4573945; doi:10.1186/s12989-015-0103-7)
Supplement: Additional file 7: — Blood and Plasma. 2-Way ANOVA with EHC-93 (0, 5, 50 mg/m3) and Ozone (0, 0.4, 0.8 ppm) as factors immediately after exposure. (DOCX 16 kb) [file 12989_2015_103_MOESM7_ESM.docx]

Additional File 7. Table: Blood and Plasma. 2-Way ANOVA with EHC-93 (0, 5, 50 mg/m^3^) and Ozone (0, 0.4, 0.8 ppm) as factors immediately after exposure.

| **Endpoint** | **Significant Effects** | **P** | **Tukey (p<0.05)** |
| --- | --- | --- | --- |
| Methemoglobin | Ozone | p=0.009 | 0.4 vs 0.8 ppm O_3_ |
| Sulfhemoglobin | NS |  |  |
| Carboxyhemoglobin | NS |  |  |
| Oxyhemoglobin | NS |  |  |
| Plasma m-Tyrosine | NS |  |  |
| Plasma o-Tyrosine | Ozone x EHC-93 | p=0.040 | 0 vs 0.4 ppm O_3_ within 50 mg/m^3^ EHC-93 |
| Plasma p-Tyrosine | EHC-93 | p=0.010 | 0 vs 50 mg/m^3^ EHC-93 |
| Plasma 3-Nitrotyrosine | EHC-93 | p=0.035 | 0 vs 50 mg/m^3^ EHC-93 |
| Plasma 3‑Nitrotyrosine/L‑DOPA | Ozone | p=0.029 | 0.4 vs 0.8 ppm O_3_ |
| Plasma BET-1 | Ozone x EHC-93 | p<0.001 | 0 vs 0.8 ppm O_3_ within 0 mg/m^3^ EHC-93  0 vs 0.8 ppm O_3_ within 50 mg/m^3^ EHC-93  0 vs 50 mg/m^3^ within 0 ppm O_3_  5 vs 50 mg/m^3^ within 0 ppm O_3_  0 vs 50 mg/m^3^ within 0.8 ppm O_3_ |
| Plasma ET-1 | Ozone x EHC-93 | p<0.001 | 0 vs 0.8 ppm O_3_ within 0 mg/m^3^ EHC-93  0.4 vs 0.8 ppm O_3_ within 0 mg/m^3^  0 vs 0.4 ppm O_3_ within 50 mg/m^3^  0 vs 0.8 ppm O_3_ within 50 mg/m^3^ EHC-93  0 vs 50 mg/m^3^ within 0 ppm O_3_  5 vs 50 mg/m^3^ within 0 ppm O_3_  0 vs 50 mg/m^3^ within 0.8 ppm O_3_ |
| Plasma ET-2 | NS |  |  |
| Plasma ET-3 | NS |  |  |
| Ratio ET-1/BET-1 | Ozone x EHC-93 | p=0.006 | 0 vs 0.8 ppm O_3_ within 0 mg/m^3^ EHC-93  0.4 vs 0.8 ppm O_3_ within 0 mg/m^3^ EHC-93  0 vs 50 mg/m^3^ within 0 ppm O_3_  0 vs 50 mg/m^3^ within 0.8 ppm O_3_ |
| Ratio ET-1/ET-3 | Ozone x EHC-93 | p=0.001 | 0 vs 0.8 ppm O_3_ within 0 mg/m^3^ EHC-93  0.4 vs 0.8 ppm O_3_ within 0 mg/m^3^ EHC-93  0 vs 0.8 ppm O_3_ within 50 mg/m^3^ EHC-93  0 vs 50 mg/m^3^ within 0 ppm O_3_  0 vs 50 mg/m^3^ within 0.8 ppm O_3_ |
